# Barriers and facilitators to development and implementation of a rural primary health care intervention for dementia: a process evaluation

**DOI:** 10.1186/s12913-019-4548-5

**Published:** 2019-10-17

**Authors:** Debra Morgan, Julie Kosteniuk, Megan E. O’Connell, Andrew Kirk, Norma J. Stewart, Dallas Seitz, Melanie Bayly, Amanda Froehlich Chow, Valerie Elliot, Jean Daku, Tracy Hack, Faye Hoium, Deb Kennett-Russill, Kristen Sauter

**Affiliations:** 1 Canadian Centre for Health & Safety in Agriculture, University of Saskatchewan, 104 Clinic Place, Saskatoon, SK S7N 2Z4 Canada; 2 Department of Psychology, University of Saskatchewan, Arts 182, 9 Campus Drive, Saskatoon, SK S7N 5A5 Canada; 3 Department of Medicine, Neurology Division, University of Saskatchewan, Saskatoon, SK Canada; 4 College of Nursing, University of Saskatchewan, 104 Clinic Place, Saskatoon, SK Canada; 5 Department of Psychiatry, Providence Care - Mental Health Services, Queen’s University, 752 King Street West, Kingston, ON K7L 4X3 Canada; 6 Saskatchewan Health Authority, Kipling, SK Canada; 7 Cumming School of Medicine and Hotchkiss Brain Institute, University of Calgary, 2919 Health Sciences Centre, 3330 Hospital Drive NWt, Calgary, AB T2N 4N1 Canada

## Abstract

**Background:**

With rural population aging there are growing numbers of people with dementia in rural and remote settings. The role of primary health care (PHC) is critical in rural locations, yet there is a lack of rural-specific PHC models for dementia, and little is known about factors influencing the development, implementation, and sustainability of rural PHC interventions. Using a community-based participatory research approach, researchers collaborated with a rural PHC team to co-design and implement an evidence-based interdisciplinary rural PHC memory clinic in the Canadian province of Saskatchewan. This paper reports barriers and facilitators to developing, implementing, and sustaining the intervention.

**Methods:**

A qualitative longitudinal process evaluation was conducted over two and half years, from pre- to post-implementation. Data collection and analyses were guided by the Consolidated Framework for Implementation Research (CFIR) which consists of 38 constructs within five domains: innovation characteristics, outer setting, inner setting, individual characteristics, and process. Data were collected via focus groups with the PHC team and stakeholders, smaller team workgroup meetings, and team member interviews. Analysis was conducted using a deductive approach to apply CFIR codes to the data and an inductive analysis to identify barriers and facilitators.

**Results:**

Across all domains, 14 constructs influenced development and implementation. Three domains (innovation characteristics, inner setting, process) were most important. Facilitators were the relative advantage of the intervention, ability to trial on a small scale, tension for change, leadership engagement, availability of resources, education and support from researchers, increased self-efficacy, and engagement of champions. Barriers included the complexity of multiple intervention components, required practice changes, lack of formal incentive programs, time intensiveness of modifying the EMR during iterative development, lack of EMR access by all team members, lack of co-location of team members, workload and busy clinical schedules, inability to justify a designated dementia care manager role, and turnover of PHC team members.

**Conclusions:**

The study identified key factors that supported and hindered the development and implementation of a rural-specific strategy for dementia assessment and management in PHC. Despite challenges related to the rural context, the researcher-academic partnership was successful in developing and implementing the intervention.

## Introduction

Rural population aging is an international phenomenon [1]. With a higher proportion of seniors in rural compared to urban areas, and increasing risk of dementia with age [2], there are growing numbers of people with dementia living in rural and remote settings [3]. The role of primary health care (PHC) is critical in rural settings because of lack of access to specialist services [2, 4]. Alzheimer Disease International [5] notes that the current specialist model of service delivery for dementia is not feasible or sustainable due to inadequate numbers of specialists, particularly in resource-poor settings. An alternate model where PHC has a central role is more sustainable and more appropriate because care coordination, a best practice in dementia care, is a key function of PHC [5]. Yet current models of PHC for dementia are primarily urban based and may not be generalizable to rural settings, because they do not specifically address the geographic and service delivery challenges in sparsely populated low-resource settings [6, 7]. Comprehensive integrated models of PHC for dementia are associated with better outcomes [8], but there is a lack of rural-specific strategies for implementing these approaches. As well, little is known about the factors that enable and impede the implementation and sustainability of PHC in rural settings [7].

The growing body of knowledge in implementation science has found that the uptake and sustained use of research evidence in practice is more likely when new programs are developed in collaboration with community partners and tailored to the local context [9]. However, a recent scoping review of implementation research in dementia care [10] identified a lack of robust evidence to inform dissemination and implementation of evidence-based dementia care. Recommendations for research included use of theories to identify the barriers and facilitators of desired change, and investigation into how to successfully implement best practices in dementia care, especially in PHC settings [10].

For over 20 years the Rural Dementia Action Research (RaDAR) Program has focused on addressing issues in rural dementia care [11]. In 2004 we implemented a University-based one-stop interdisciplinary Rural and Remote Memory Clinic, to improve access to specialist diagnosis and management of complex, atypical dementias [12, 13]. The fact that over 60% of clinic referrals are for Alzheimer Disease, which Canadian guidelines recommend be managed in PHC settings [14], suggests that rural PHC providers are looking for support in making these diagnoses. Our research has identified diagnostic delays [15] and challenges in the provision of dementia care in rural PHC settings [16–18]. This paper reports the findings of a process evaluation conducted over two and half years, to inform the development and implementation of a rural PHC intervention, and identify barriers and facilitators to developing, implementing, and sustaining the intervention in a rural PHC team.

## Methods

**Fig. 1:**
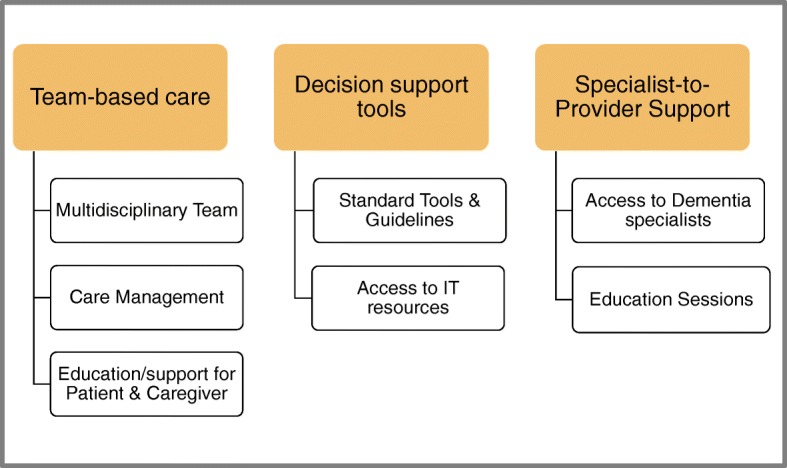
Rural Primary Health Care Model for Dementia

To provide an evidence-based foundation for the rural PHC intervention we created the Rural PHC Model by identifying strategies that were associated with better outcomes in an extensive scoping review of international literature on interventions for community-based dementia care [8]. Seven strategies that were found to be associated with better outcomes were incorporated into three domains in the Rural PHC Model for Dementia: team-based care, decision support tools, and specialist-to-provider support (Fig. 1). We then collaborated with a rural PHC team, using a community-based participatory research approach [19] to iteratively co-design and implement an intervention that operationalizes the model elements in ways that were feasible, effective, and sustainable within the rural context. The final version of the intervention that emerged from the co-design process involved a one-day interdisciplinary PHC memory clinic, described below. The development and implementation process was informed by published frameworks for modifying evidence-based interventions for local settings [20–23] in a 5-step approach: (1) relationship-building, (2) needs assessment, (3) identifying key elements of the intervention to be adapted, (4) iterative implementation and adaptation of the intervention, and (5) sustaining the intervention while scaling up. These steps have been described in a previous publication [24].

### Study intervention: rural primary health care memory clinic

A brief overview of the intervention that resulted from the researcher-primary health care team collaboration is provided here. More detail is provided in the results section under the relevant CFIR constructs. Prior to establishing the Rural PHC memory clinic, patients with suspected dementia were assessed by their family physician or nurse practitioner, with little involvement of other disciplines linked to the PHC site and no standardized assessment approach. Because team members were not co-located, the first iteration of the intervention involved a sequential approach, with team members taking turns conducting their assessments (in the clinic for physicians and the nurse practitioner, and in the home for the home care nurse and occupational therapist). This approach was not effective because of delays in receiving notification in the electronic medical record (EMR) system for team members who were not regular users, and the EMR did not accommodate multiple team members entering the chart at different times. The decision to adopt the memory clinic model eliminated many of these EMR functionality issues and reduced the complexity of assessing dementia as individual team members who were not co-located. The memory clinic, which is ongoing, is held every one to 2 months, with two patients and their families attending for a half-day each. Team-based follow-up appointments for ongoing management are also scheduled for the memory clinic days.

**Fig. 2:**
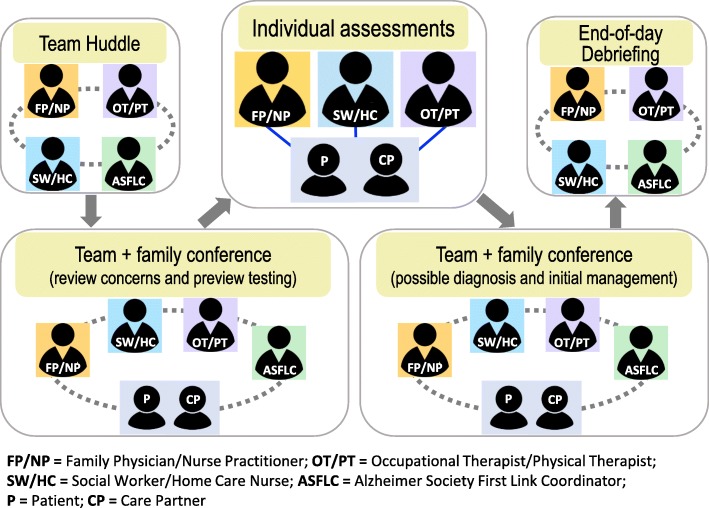
Patient Care Pathway for 1-day Memory Clinic Appointment

The care pathway for the 1-day memory clinic (Fig. 2) involves a team huddle to review the concerns leading to the referral and any previous testing, a team case conference with the patient and family, individual team members’ assessments, a team debriefing, and a final team case conference to discuss the findings with the patient and family and develop a care plan. Team-based follow-up appointments are scheduled at three to 6 months. Evidence-based decision support tools are used to guide the initial evaluation and follow-up appointments.

The *decision support* domain in the Rural PHC Model for Dementia was operationalized in the PHC memory clinic by adapting an existing decision support tool, the Primary Care – Dementia Assessment and Treatment Algorithm (PC-DATA™) [25]. The tool was originally developed for primary care physicians supported by a Dementia Care Manager and was not team-based or designed for EMR use. PC-DATA™ included visit flow sheets to guide the assessment, diagnosis, and ongoing management of dementia based on Canadian guidelines [14]. The flow sheet components were reorganized and grouped according to the expertise of the interdisciplinary team members so that each had a defined role. The original three flow sheets were streamlined into two (initial assessment and diagnosis, and ongoing management), which were then embedded in the team’s EMR system. Coordinated *team-based care* was operationalized by coordinating an interdisciplinary team (see below) to participate in the memory clinics, with roles tailored to members’ expertise. The Alzheimer Society First Link Coordinator from the region was invited to be part of the team to provide education and support to patients and family members. *Specialist-to-provider support* was provided by regular education sessions delivered in-person and by telehealth by the PC-DATA developer (DS) and RaDAR clinical specialists (AK, MEO), all members of the research team.

### Study design and setting

A qualitative longitudinal process evaluation was conducted across the pre-implementation, implementation, and post-implementation phases. The study took place in the Western Canadian prairie province of Saskatchewan, Canada (population 1 million, area of 651,000 km^2^). This research is part of an ongoing partnership between RaDAR and the Sun Country Health Region (population 60,000, area 33,329 km^2^, density 1.8 persons/km^2^_,_ 15% age 65 or older). The region has two urban centres of approximately 11,000 people, with 58% of the population living in rural areas under 10,000 population.

The study was guided by a regional Steering Committee of managers of PHC, home care, long-term care, mental health, and chronic disease care. The committee recommended one of the region’s seven PHC teams to collaborate with RaDAR to co-design and implement the intervention, before scaling up to other teams in the region. Selection criteria included presence of a champion and stability of team members, particularly physicians, since there is frequent turnover and often delays in recruitment in rural communities. The initial PHC team was located in a community of 1000 people 400 kms from the RaDAR team, where the nearest specialist services were 1.5 h away. The team (see Table 1) included three family physicians (all of whom completed their contracts before the end of the study), a nurse practitioner, an occupational therapist (the first one moved and was replaced), two home care nurses (one retired part way through the study), a PHC team facilitator (the first one went on short-term leave and was replaced), and a business/EMR manager. The physicians and nurse practitioner were based in the community’s PHC clinic; other team members were linked to the clinic but served other communities and were not co-located.

**Table 1:** Study Participants by Data Collection Strategy

Participant	Focus Group Meetings (FG)					Workgroup Meetings (WG)				Total n FGs & WG	Individual Phone Interview
	FG 1	FG 2	FG 3	FG 4	Total n FGs	WG 1	WG 2	WG 3	Total n WGs		
Primary Health Care Team Members											
Family Physician	✓	✓	✓		3				0	3	
Family Physician	✓	✓	✓		3	✓			1	4	
Family Physician	✓		✓	✓	3				0	3	
Nurse Practitioner	✓	✓	✓	✓	4	✓	✓	✓	3	7	
Occupational Therapist & Regional Manager of Therapies	✓	✓	✓	✓	4	✓	✓		2	6	✓
Occupational Therapist 1 (Aug/14-Feb/16)		✓			1				0	1	
Occupational Therapist 2 (Sept/17-Jan/18)					0			✓	1	1	
Home Care Nurse 1 (Aug/14-Jan/18)	✓		✓	✓	3			✓	1	4	
Home Care Nurse 2 (Aug/14-Feb/16)	✓	✓			2				0	2	
Alzheimer Society, Sun Country First Link Coordinator			✓		1	✓		✓	2	3	✓
Primary Health Care Team Facilitator 1 (Dec/15-Feb/16)		✓			1				0	1	✓
Primary Health Care Team Facilitator 2 (Aug/14-Jan/18)				✓	1	✓	✓	✓	3	4	
Regional Business Manager, Primary Health Care		✓	✓	✓	3	✓	✓	✓	3	6	✓
Managers											
Home Care Manager		✓	✓		2				0	2	
Manager Home Services					0	✓			1	1	
Alzheimer Society Manager		✓			1				0	1	
Community Health Services Manager	✓	✓	✓		3				0	3	
Chronic Disease Management Coordinator	✓				1				0	1	
Regional Manager, Primary Health Care Teams	✓	✓	✓	✓	4				0	4	
Regional Manager, Chronic Disease Management	✓	✓			2				0	2	
Regional Director, Mental Health and Addictions	✓				1				0	1	
Office Staff											
Medical Office Assistant/Office Staff	✓				1	✓			1	2	
Medical Office Assistant/Office Staff	✓				1				0	1	
Medical Office Assistant/Office Staff	✓				1				0	1	
Medical Office Assistant/Office Staff	✓				1			✓	1	2	
Totals	16	13	11	7	47	8	4	7	19	66	

Across the three data collection strategies, 25 unique individuals participated in the study (2 males and 23 females). Workgroup participants were a subset of focus group participants. Some individuals participated in only one event, while others participated in multiple events

**Fig. 3:**
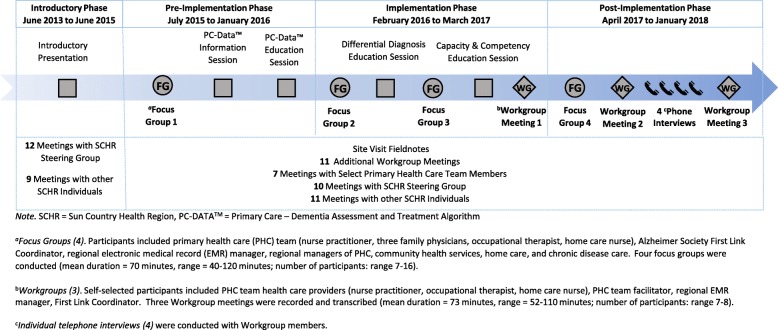
Data Collection Timeline

Figure 3 shows a timeline of study phases. The purpose of the introductory phase was to build relationships at the regional and PHC team level and conduct a regional needs assessment [24]. The pre-implementation phase was aimed at identifying gaps in the team’s current dementia care practices, assessing the implementation context, and providing education on dementia assessment, diagnosis, and management. The goal of the implementation phase was to engage all team members in iteratively developing and implementing the intervention. Post-implementation, the focus was on continued refinement of the intervention and understanding barriers and facilitators to long-term sustainability.

### Study participants

The work of co-designing the intervention was conducted through focus groups (full PHC team, key stakeholders recommended by the Steering Committee, and the researchers), and a smaller workgroup (PHC team members who self-selected to the group, and the researchers). Table 1 identifies participants involved in the three data collection strategies: the focus groups and workgroups, and individual interviews. A total of 25 unique individuals took part in the study (2 males and 23 females). Four in-person *focus groups* (7 to 16 participants) were conducted across the three implementation phases. Office staff participated initially but discontinued as the discussion became more clinical. Also, focus groups were held off site and it may not have feasible for them to leave the clinic. Three smaller *workgroup meetings* (4 to 8 participants) were held during implementation and post-implementation to facilitate more frequent communication, resolving implementation challenges and facilitating implementation. Post-implementation, four *telephone interviews* were conducted with workgroup members who were still with the team at that stage. Physicians were in the community on 3-year contracts, which did not align with this 5-year study. At post-implementation the contracts of all three physicians had ended and they left the community. A temporary locum and one new physician had been recruited but not yet oriented to the intervention.

### Data collection

Data collection and analyses were guided by the Consolidated Framework for Implementation Research (CFIR) [26]. The CFIR provides a structure for understanding barriers and facilitators to successful implementation in specific settings, enhancing fit with the local context, and informing dissemination to other settings. The CFIR consists of 38 constructs organized into five domains: (1) innovation characteristics (key attributes influencing implementation), (2) outer setting (the economic, political, and social context within which the organization resides), (3) inner setting (the structural, political, and cultural contexts in which the implementation process occurs), (4) individual characteristics (the cultural, organizational, and professional norms and interests of those involved with the intervention and implementation), and (5) implementation process (the strategies and individuals involved in the change process) [26]. The framework can be used at all implementation phases [27]. In this study the CFIR was used to inform development of interview guides (pre-implementation); identify implementation barriers and strategies to overcome them, tailor the intervention, and refine implementation strategies (implementation); and identify constructs that were most salient to successful implementation (post-implementation).

The data collected in each phase is shown in Fig. 3. Structured CFIR interview guides, developed by the RaDAR team based on CFIR domains and constructs [26], were used for the pre-implementation focus group (Additional file 1) and post-implementation telephone interviews (Additional file 2). No other structured guides were used because the main focus of the focus groups and workgroups was actively designing the intervention and making modifications as needed. The focus groups, workgroups, and interviews were audio-recorded, transcribed verbatim, and checked for accuracy. Transcripts were anonymized to preserve participant anonymity. Additional contextual data included researcher site visit fieldnotes, emails with participants, and meeting notes with members of the PHC team, workgroup, and Steering Group.

### Data analysis

This study used a deductive approach to analysis using the CFIR domains and constructs as an a priori coding framework [28–30] and an inductive analysis to identify barriers and facilitators to development and implementation [31]. Damschroder et al. [26] note that constructs should be assessed for salience and adapted for the particular context. Without prior knowledge of which specific constructs would be most relevant, we assessed for all 38 original CFIR codes. Sustainability, which has been identified as a gap in the CFIR [32], was added because of our interest in long-term sustainability of the intervention. Examining sustainability throughout an initiative (prospective assessment) is useful for embedding an initiative into an organization and enhancing buy-in [33]. Planning for sustainability in the implementation phase by assessing the fit between the context and the intervention helps to ensure that the intervention can be maintained over time [34].

An analytic team of five investigators (DM, JK, MB, VE, AFC) conducted all analysis steps except where noted. In the first step, operational definitions tailored to the study were defined for each CFIR domain and construct (Additional file 3). As coding progressed, definitions were refined to improve coder consistency; at each step previously coded data were re-coded using the revised definitions. Three team members [DM, JK, VE] independently coded one transcript to test the definitions. At step two, coding pairs independently coded each of the 11 transcripts, using paper versions to manually code. Content analysis [28] was used analyze the content of transcripts and apply CFIR codes. Coding pairs resolved coding differences, followed by a team meeting to discuss discrepancies and agree on revised operational definitions. The transcripts were then imported into the CFIR NVivo Project Template [35] and the codes applied. In step three, three transcripts (one from each phase) were selected and code reports generated from NVivo with all the data segments for each code, organized by CFIR domain. These collated data were reviewed independently by all team members for consistency in application of the codes, followed by meetings to resolve disagreements, further refine definitions, and review instances of double-coding with the aim of applying only one code where possible. In step four, all team members independently reviewed the coding in the remaining eight transcripts to ensure that final definitions were applied. In step five, an inductive analysis was conducted to identify implementation barriers and facilitators. Rigor was supported by the longitudinal data collection, prolonged stakeholder engagement, triangulation of data from multiple sources and stakeholders, documentation of all communication with participants, and iterative team-based coding and analysis [31, 36].

## Results

Fourteen constructs within the five domains emerged as important to the development and implementation process, and intervention sustainability. The step-wise development-implementation process was so iterative and incremental that it is not possible to separate factors influencing development vs. implementation of the memory clinic intervention. The domains of innovation characteristics, inner setting, and process were most influential. Key constructs within each domain are reported below with illustrative quotations. A summary of facilitators and barriers to development and implementation of the rural PHC model are reported by CFIR domain in Table 2.

**Table 2:** Facilitators and Barriers of the Rural Primary Health Care Memory Clinic Intervention using the Consolidated Framework for Implementation Research

CFIR Domain and Constructs	Barrier	Facilitator
**Innovation Characteristics**		
Relative Advantage		Evidence-based flow sheets provided standardized assessment tool for all team members Team approach and standardized tools increased team members’ confidence in providing care without having to refer all patients Team members felt valued for their unique contribution to assessment and management Benefits to families including giving them a voice, providing direction, supporting future planning, connecting with services, avoiding crises
Trialability	Small-scale, iterative implementation and testing of the EMR flow sheets hampered by time intensiveness of modifying the EMR	Despite EMR challenges, the intervention could be implemented on a small scale to assess feasibility and iteratively test modifications to improve fit to context
Complexity	Having the assessment flow sheets in the EMR was critical to implementation, but having multiple team members accessing the EMR created challenges that had to be resolved	The EMR created implementation challenges but it also reduced complexity by supporting team-based care and access to evidence-based decision support tools
**Outer Setting**		
Needs and resources of those served by the innovation		Team members concerned about unmet needs of patients and families with usual care approach; late diagnosis and lack of support contributed to crisis situations Team approach and case conference facilitates discussion with family about services and planning for future needs Alzheimer Society participation in the memory clinics may increase use of supports by developing a relationship at time of diagnosis
External policy and incentives	Home care used a different EMR system that was not compatible with the PHC team EMR Policy of not funding licences for home care nurses to access the PHC team EMR Dementia not included in provincially funded incentive program for family physicians to use evidence-based tools with chronic disease patients	Improving access to primary health care teams is a priority for Ministry of Health
**Inner Setting**		
Networks and Communications	Not all team members had EMR access initially Not all team members co-located Busy clinical schedules made it difficult to schedule meetings to develop and implement the clinic Researchers did not have direct communication with physicians	The team’s facilitator was critical to communication among team members and with the researchers. They could view calendars and book team members into meetings. Their formal role in team development benefited implementation by supporting communication. The memory clinic EMR was set up to accommodate access to the patient record by all team members
Tension for change (Implementation Climate sub-construct)		Dissatisfaction with current approach to care; uncertainty about assessment process led to late diagnosis, often precipitated by a crisis situation Silo approach and lack of care coordination was less effective than a collaborative team approach Discussion about driving capacity in the team case conference removed the burden from one team member and reinforced the message to patients and families
Compatibility with existing workflows and processes (Implementation Climate sub-construct)	Team physicians perceived the team-based memory clinic model as inconsistent with their usual iterative approach to assessment Physicians’ involvement with other chronic disease case was less intensive; other team members managed most of the assessments and communication with patients and families.	Some team members were already experimenting with involving the Alzheimer Society and home care in a case conference when dementia suspected
Leadership engagement (Readiness for Implementation sub-construct)		The support and active engagement of leaders was critical to ensuring adequate resources for the intervention, communicating the importance of the intervention, and giving permission to team members to participate
Available resources (Readiness for Implementation sub-construct)	Workload was a challenge to participation in the memory clinic for all team members Lack of personnel such as Dementia Care Managers to support the clinic and ease workload for team members Challenges in recruitment and retention of family physicians was a major barrier	The team facilitator and EMR manager were committed to the project and supported implementation despite workload issues The primary health care site had multiple allied health care providers linked to the site who could be accessed to participate in the memory clinic intervention
Access to Knowledge and information (Readiness for Implementation sub-construct)		Few educational opportunities were available prior to the intervention; education by RaDAR specialists and PC-DATA™ developer helped build confidence in assessment and management Observing in the University-based interdisciplinary specialist memory clinic run by the RaDAR team inspired the rural PHC team to adopt the one-day clinic vs. the initial sequential approach Workgroup meetings with the researchers, RaDAR Handbook^a^, and tools embedded in the EMR were helpful
**Characteristics of Individuals**		
Self-efficacy		Team members’ self-efficacy and ownership of the intervention increased over the study. Growing confidence and feelings of contributing to improved outcomes for patients and families motivated continued involvement
**Process**		
Champions		Key individuals within the team who facilitated implementation were the nurse practitioner, PHC facilitator, and EMR manager
External change agents	Absence of a formally appointed internal facilitator	Participants identified the RaDAR researchers and PC-DATA™ developer as supporting implementation by providing education and working closely with the team at all stages to facilitate implementation and maintain momentum
**Innovation Sustainability**	Physician turnover Lack of process to engage and orient new team members, especially physicians, to the flow sheets and memory clinic processes	Continued contact with the researchers Consistent leadership in the region Increased community awareness of the memory clinic

^a^The RaDAR Handbook was created by the team to consolidate the tools and resources developed to support the one-day PHC memory clinic (e.g., PC-DATA™ flow sheets for the initial evaluation and monitoring/follow-up, templates for letters to confirm appointments and summarize outcomes of the initial evaluation for patients and families, work standards to guide clinic processes, PC-DATA™ Educational Manual, and scripts to support PHC team members in discussing topics such as driving and communication a diagnosis). The Handbook was available online and in hard copies distributed to all team members

### DOMAIN 1: innovation characteristics

This was a key domain, with three constructs emerging as important to implementation: relative advantage, trialability, and complexity.

#### Relative advantage

Team members reported that the team-based standardized evidence-based assessment flow sheets helped them provide better care by providing a template or guide to assessment steps. They reported increased confidence and feeling empowered to provide better care without having to refer all patients to specialists.

*“We knew that the dementia part of our patients was important and assessment of that—we just didn’t quite know how to put all together and bring everybody together. So that’s been huge.” (Manager, Post-implementation, Focus Group 4)*

*“The providers did the best they could in their appointments … [but] they didn’t feel like it was very standardized so they wanted a process for when a patient complains of cognitive difficulties, what exactly do we do, what are the steps that we take.” (PHC Team Member, Post-implementation, Telephone Interview)*

The team approach also allowed team members to contribute their individual disciplinary skills to the assessment. They felt valued by other team members and in turn appreciated other team members’ roles. The format of the one-day clinic allowed team members to discuss their findings and learn from each other, thus increasing their confidence in the diagnosis and treatment plan. The synergies of working together resulted in better care, which was rewarding.

*“As a provider I feel much more confident dealing with these people AND because I know there’s a team that backs me up too.” (PHC Team Member, Post-implementation, Focus Group 4)*

Team members identified benefits to families of the interdisciplinary memory clinic model, which included giving them a voice, providing direction, and enabling them to plan for the future and avoid crises. Team members had seen the negative consequences of not connecting patients and families with available supports early on and were relieved that this gap was filled by Alzheimer Society First Link coordinator with specific skills in assessing and supporting patients and families.

*“I think too the fact that we’re together and we all come from maybe a little bit of a different slant, but we were really speaking the same language to the family. I think they get a really good overall picture of what the issues are and what the plan could be. That’s part of it, that it looks like a really concerted effort as far as providing quality care for the clients.” (PHC Team Member, Post-implementation, Workgroup Meeting 3)*

*“And I think the family felt like they had a voice. And once they had that knowledge given to them, I mean you can just see it sink in and you can just see the wheels turning and I knew that they had questions and so before we’re over [case conference], we all went around the room and asked [the family] ‘what else do you have to say? Was there a question that you thought of?.... the family yesterday said ‘where’s the plan? What’s the first step that we do?’”(PHC Team Member, Post-implementation, Workgroup Meeting 3)*

#### Trialability

Ability to test the innovation on a small scale and undo the implementation if needed was important because it allowed the team to iteratively develop, test, and modify strategies to implement team-based care and the decision support tools to fit their context. From the outset the team determined that implementation and sustainability depended on the PC-DATA™ visit flow sheets being integrated into the EMR system. They first tested paper versions of the revised visit flow sheets to assess acceptability of the re-organized content before creating a trial EMR version. Once in the EMR, however, further iterative testing was hampered by the functionality of the EMR platform, which was not easily modifiable once forms were created (see Complexity construct).

*“Instead of putting all three flow sheets in what if I try a small sampling of the flowsheets … I’m actually in [town] Friday, and so I can show the providers some of the templates I’ve developed, and some of the ways some of the things work … Kind of just put in a trial flowsheet with some of the information, see how it looks, and we can tweak it?” (Manager, Implementation, Focus Group 2)*

#### Complexity

Complexity was defined as the perceived difficulty of implementing the intervention, and was related to multiple intervention components and issues with the EMR. Having the PC-DATA™ flow sheets in the EMR was critical because the EMR was standard practice in the clinic and the flow sheets helped to operationalize several components of the RaDAR PHC Model, including coordinated team-based care and access to evidence-based decision support tools. The EMR also allowed functions such as ability to download and print embedded scales, and links to resources and referral forms. However, having multiple team members completing different sections in the EMR at different times required many discussions about how to document this information. Given that the chart is a legal document there was concern that it be clear who had charted which sections. Technical questions arose about bringing forward previously entered data into the flow sheet. Completed scales were scanned and uploaded so that drawings and responses to individual items could be tracked over time but the files were difficult to find in the EMR.

*“There is another barrier. When it is scanned, what happens is that based on the label that is put on top, it ends up in the wrong place, so you are looking for something and you don’t find it … so when I’m going to review the past thing I may end up not finding the clock that the patient has done, and I think ‘okay it’s not done.’” (PHC Team Member, Implementation, Focus Group 2)*

Planning the one-day memory clinics created complexity because it required the PHC team to coordinate their schedules, contact patients and families, and book clinic space. However, it reduced the complexity of EMR issues linked to the sequential assessment approach and meant that team members were co-located on clinic days and face-to-face for the full assessment process.

### DOMAIN 2: outer setting

#### Needs and resources of those served by the innovation

Patient and family needs were a major implementation driver. Most team members supported the project because they had long-standing concerns about unmet needs and believed that the intervention could help with earlier diagnosis and maintaining the person with dementia in the community.

*“It’s just really exciting to think of the possibilities, and across the continuum of care, the early diagnosis is really important, and how we can help support our families as long as we can at home.” (PHC Team Member, Pre-implementation, Focus Group 1)*

Team members reported that late diagnosis and lack of support often contributed to crisis placement in long-term care. The coordinated team approach facilitated discussions about available services and planning for future needs. Team members anticipated that by including the Alzheimer Society First Link Coordinator in the clinic assessment the family would be more likely to accept supports in the future.

*“The families were just so appreciative of getting together at the end of our all our testing and talking about it and including their family member … . they’re going home with something to think about and some ideas.” (PHC Team Member, Post-implementation, Workgroup Meeting 3)*

#### External policy and incentives

The regional policy of not funding EMR licenses for home care nurses due to the high cost restricted their ability to participate in the team-based approach. The nurses completed paper versions of the flow sheets, but the issue then was how the data would get entered into the EMR as office staff did not have access to the patient records. Another policy barrier was obtaining permission for nurses to assess patients who were not existing home care clients. After revisiting these problems at every PHC team meeting, managers participating in the study successfully advocated to senior leadership for these changes, on the basis that allowing home care nurses to participate fully in the project supported comprehensive use of the existing PHC team to improve patient care, which was important in rural settings with limited access to specialist resources.

“I think what’s important to revisit as well is the ability to use our team to a comprehensive level … if somebody’s not a home care client, that shouldn’t preclude the team being able utilize someone like [home care nurse] to help with the assessment.” (Manager, Implementation, Focus Group 3)

Another policy barrier was the fact that dementia was not one of the few conditions included in the provincial chronic disease quality improvement program, which mandated implementation of assessment templates for four conditions and provided financial incentives for physicians to complete the templates.

### DOMAIN 3: inner setting

Along with characteristics of the intervention, the inner setting of the PHC team was a key domain influencing implementation.

#### Networks and communications

Frequent formal and informal communication among the PHC team members was needed to develop and implement the intervention but not all team members were based in the same community. The EMR system was the most efficient way of communicating but the home care nurse initially lacked access and the occupational therapist lost access if she did not log in regularly. The collaborative approach to developing the intervention necessitated regular meetings between the researchers and team members. In-person meetings were preferable and held as often as feasible but due to long distance travel for the researchers and lack of co-location of PHC team members, videoconferencing and teleconferences were used to facilitate communication. Finding meeting times was challenging due to team members’ busy schedules and clinical demands, and the fact that some team members also served other communities on certain days of the week.

*“They [PHC team members] are going about their daily work and daily duties and business which is seeing their patients. Time, scheduling, coordinating to get together for meetings is always a barrier but is overcome when everyone is aware of the benefits—that it will improve their seeing patients and the care they provide.” (PHC Team Member, Post-implementation, Telephone Interview)*

Since the researchers did not have direct communication with the physicians, the support of the PHC facilitator and regional business manager/EMR manager was critical. Although not co-located in the PHC clinic they were on-site regularly and could schedule meetings in team members’ calendars. Because their roles included quality improvement and supporting collaborative practice, they were knowledgeable about team processes and organizational structures, which facilitated implementation.

#### Implementation climate

Two of the six sub-constructs emerged as relevant: tension for change and compatibility with current work patterns.

##### Tension for change

Dissatisfaction with the current approach to care for patients with dementia and their families was a strong motivation for PHC team members to develop a new model of care that was evidence-based. Previously, uncertainty about the assessment process led to later diagnosis of dementia, often precipitated by a crisis situation resulting in long-term care placement that may have been avoided with earlier intervention.

*“I was all for it just because I had been seeing lots of people with pre-dementia or dementia and I had seen some less-than-desirable effects from people falling through the cracks because of … practitioners not knowing what to do, or where to go from here.” (PHC Team Member, Pre-implementation, Focus Group 1)*

Patients were assessed by their physician or nurse practitioner, who then referred some patients to other care providers and received results from these assessments, but there was little interaction between providers or coordination of care. This parallel assessment approach was less effective than working together.

*“We’re seeing people in silos; we get a referral, we write a report, we send it back and there is never any discussion between the different people and the family on what we should do.” (PHC Team, Implementation, Focus Group 2)*

Pre-implementation, occupational therapists, who were delegated responsibility for assessing driving capacity, felt “thrown under the bus” when patients and family members became upset when driver’s licenses were revoked. With the one-day clinic, driving issues were discussed as a team in the case conference with the patient and family. With diagnostic assessment occurring earlier in the disease process, driving issues could be discussed before cessation became necessary, and some patients decided to stop driving voluntarily, which eased the situation for patients, families, and the team.

*“The whole driving thing, that’s much better … it’s definitely something we can talk about now, rather than just me having to send that in to [insurance company]. It’s better if we can make a group decision.” (PHC Team Member, Post-implementation, Focus Group 4)*

##### Compatibility with existing workflows and processes

Although PHC team physicians were salaried and could book 30-min appointments, compatibility of the comprehensive dementia assessment and case conferences with their workload and scheduling patterns was a concern. They typically used an iterative approach when dementia was suspected, which was perceived as incompatible with the one-day memory clinic model. Physicians suggested that the approach used with other chronic conditions could work, where the physician attended the assessment briefly and most of the assessment and discussion with families was completed by other team members.

*“When these forms were not there, what we did was this. We started to do the physical, history, asking for bloodwork. I waited for the results of the bloodwork and then if there was any imaging needed … . [it took] two to three visits.” (PHC Team Member, Implementation, Focus Group 3)*

The ability to draw on a number of health care disciplines associated with the PHC site was identified as compatible with the team-based care domain in the Rural PHC Model.

*“I think for multidisciplinary team, I think we’re pretty strong with that; there’s many people that can be involved, that we can pull in. We have OT, we have home care, we have practitioners.”* (PHC Team Member, Pre-Implementation, Focus Group 1)

#### Readiness for implementation

All three readiness subconstructs (leadership, resources, and access to knowledge and information) played a role in implementation.

##### Leadership engagement

The support and active involvement of leaders was an important facilitator. The early engagement of the Steering Committee ensured that high-level regional leaders on the committee were aware of the region’s commitment to the study. The interdisciplinary clinic brought together health care providers from departments outside PHC where team-based care may not have been as engrained, thus support from their managers to participate in research team meetings and the one-day clinic was not automatic for all team members.

*“These meetings do take time from the day, so just to have the support from managers and supervisors you know, to attend the meetings, to be part of the team, and to help develop [the intervention] would definitely [help] them to continue.” (Manager, Post-implementation, Telephone Interview)*

The regional managers who actively participated in all development and implementation stages were important because they had first-hand knowledge of implementation barriers and could help address them, and advocate for needed resources. Participants suggested that all managers and leaders in the region should be made aware of the intervention, even if not actively involved, so they could support implementation by removing barriers and giving staff permission to be involved.

*“When we don’t have the luxury of something like [specialist Rural and Remote Memory Clinic] and that’s all your focus is, which is awesome, where this team is all trying to put the bits together as a team for the patient. I think we need to reach out and think outside the box and think how can we do this.” (Manager, Implementation, Focus Group 3)*

*“You have to have somebody that says ‘yes we are willing to do that.’ Or else your project is dead in the water.” (PHC Team Member, Post-implementation, Telephone Interview)*

##### Available resources

Access to adequate human resources was critical to implementation. Workload issues were a challenge for all team members. The implementation would not have been possible without the EMR manager’s time and commitment to creating the initial EMR flow sheets and modifying them over time. The team PHC facilitator was an essential resource in communicating with team members, scheduling focus group and workgroup meetings, and helping develop resources such as work standards outlining memory clinic procedures and scripts to guide conversations on topics such as communicating a diagnosis and driving cessation. By being actively involved in all research activities they were aware of implementation challenges and could help address them.

*“The facilitators … are all phenomenal at what they do and really they do a lot of the background work that I don’t think a lot of people even recognize.” (PHC Team Member, Post-implementation, Telephone Interview)*

Another challenge was the lack of support personnel such as Dementia Care Managers (a component of the original PC-DATA™ pilot [25]), and occupational therapy resources were limited due to ongoing challenges in recruitment and retention. All three physicians completed their contracts while the study was in progress and delays in recruitment led to additional workload for the remaining physicians and nurse practitioner.

##### Access to knowledge and information

Education about dementia assessment and management were important in building team members’ confidence in conducting the memory clinics. Because education is an element of the Rural PHC model and there were few other opportunities available, RaDAR specialists and the PC-DATA™ developer (members of the research team) provided regular education sessions. Most were delivered by telehealth videoconferencing, and were well attended and received.

*“The researchers have been awesome the whole time. And they were great at the beginning, great support, great in communicating, a nice balance of visiting in person and over the phone, in communicating, so they were there, and then … the providers could identify [RaDAR specialists] to help them network and to be able to provide better care … somebody that’s not providing the care but was involved in all the meetings so I think that was good.” (PHC Team Member, Post-implementation, Telephone Interview)*

A number of PHC team members spent a day observing in the interdisciplinary specialist Rural and Remote Memory Clinic at the researcher’s (DM) university, with travel support from the RaDAR project. Seeing the roles of specialist team members and how the clinic day was structured influenced their decision to adopt the one-day model in their PHC team. The RaDAR project also supported travel to attend the annual RaDAR Rural Dementia Summit [37] and national dementia conferences.

*“A lot of the education that we’ve had over the couple of years has been really, really good … I was able to spend a day at the [specialist Rural & Remote] dementia clinic and that was really good. So you take little bits of all that you learn and you can apply them to your setting.” (PHC Team Member, Post-implementation, Focus Group 4)*

Other sources of information that facilitated implementation included the regular workgroup teleconferences between the PHC team and researchers, the RaDAR Handbook that collated all the clinic resources, and tools embedded in the EMR, such as the standardized tests and scoring guidelines.

### DOMAIN 4: characteristics of individuals

#### Self-efficacy

Stakeholder self-efficacy and ownership of the intervention increased over the phases of the research and reinforced the perceived value of the memory clinics. Prior to the intervention team members lacked complete confidence in their individual abilities to assess and manage dementia, but with the tools, resources, and team approach in place they were excited to see how their particular professional skills could make a unique contribution to improving care.

*“I think it’s influenced it [care] huge, just to have those tools, and the kind of process by which to follow … we had the misconception that everybody had to have a scan and everybody had to see a neurologist … I think it’s accomplished what I think the initial thing was about -- building capacity. It’s helped you know give us the tools and give us the confidence that yeah, we can do that.” (PHC Team Member, Post-implementation, Telephone Interview)*

The team took ownership of the intervention by shaping it to their specific context, conceiving the idea of a one-day memory clinic model in their community, and then operationalizing it. The first clinic was successful and they felt empowered to continue. However they also noted that they did not have the resources of the specialist Rural and Remote Memory Clinic.

*“We don’t have the capabilities you have at the Rural and Remote Memory Clinic. We just have what we have.” (PHC Team Member, Implementation, Focus Group 2)*

*“Very beneficial. I loved it. It was a good day.” (PHC Team Member, Post-implementation, Workgroup Meeting 2)*

*“So I think it’s going very well and it’s just really positive.” (PHC Team Member, Post-implementation, Workgroup Meeting 3)*

### DOMAIN 5: process

#### Engaging

Engaging and retaining key individuals was critical to successful development and implementation, particularly those identified as champions and external change agents.

Champions included individuals within the PHC team who moved the implementation forward. The nurse practitioner was consistently identified as a key champion, playing a leadership role through her passion, knowledge of the community, and role as a front-line health care provider.

*“She [nurse practitioner] was really instrumental after [PC-DATA™ developer] gave us the [flow sheet] document and we were kind of living with it and she would be the one who could say ‘can we make these changes with this, I think it would work better if we did this.’ And then she was the one who would go to the other team members and say ‘okay we need to use this, this is how it’s working’ … . definitely the champion right in the clinic.” (Manager, Post-implementation, Telephone Interview)*

The EMR manager championed the intervention by creating and iteratively revising the flow sheets based on feedback from team members, and supporting them in using the EMR. The PHC facilitator was also identified as a champion by coordinating meetings and acting as liaison between the PHC team and researchers.

*“She [PHC team facilitator] has been very valuable … she helps organize things, she helps write things up … I think if you didn’t have that then it would be very daunting.” (PHC Team Member, Post-implementation, Telephone Interview)*

External Change Agents were defined as those outside the health region who facilitated the implementation. In the absence of a facilitator appointed by the health region, participants identified the RaDAR researchers and PC-DATA™ developer as key to supporting implementation by working closely with the PHC team to adapt the flow sheets and develop team processes, providing education to build capacity, and facilitating the implementation process.

#### Innovation sustainability

Physician turnover was identified as a major barrier to sustainability, as was the lack of a process for orienting new team members. New PHC team members had so much to learn that making time to orient them to the clinic and EMR flow sheets was difficult. Participants indicated that orientation for all new members should be done immediately on hire and the intervention presented as standard practice, and be the responsibility of supervisors rather than fellow team members, who have their own clinical responsibilities. Engaging new physicians was difficult because there were no physician supervisors knowledgeable about the intervention. To sustain the intervention, participants recommended continuing the connection with the research team, keeping all managers involved and informed, having consistent leaders to ensure ongoing support, and increasing community awareness of the service.

## Discussion

This study addresses the gap in evidence to inform successful development and implementation of evidence-based dementia care in PHC [10] with a focus on rural settings. The extended engagement process with one PHC team over two and a half years allowed us to explore implementation barriers and facilitators from pre- to post-implementation. Despite implementation challenges, the researcher-community collaboration was successful in co-designing and implementing a best practice intervention of rural PHC for dementia. This outcome supports the growing evidence that the active engagement of stakeholders and shared decision-making in all phases of the process, which allows tailoring for specific populations, is key to translation of new scientific evidence in real world practice settings [38]. All CFIR domains were found to influence development and implementation, although three (innovation characteristics, inner setting, and process) were most important to development and implementation.

*Relative advantage, trialability* and *complexity* were most the relevant **innovation characteristics.** The team identified numerous benefits of the clinic over current practices, for patients, families, and team members. Trialability was hampered by the difficulty of embedding the PC-DATA flow sheets in the EMR system and iteratively modifying the flow sheets to accommodate team-based dementia diagnosis and management. The multiple components of the intervention had to be designed and implemented, adding to complexity. Limitations in function of the EMR platform for team-based care and the reality that not all team members had EMR access made trialing and implementing the intervention more complex and time-consuming than expected. Similar findings were reported by Warner et al. [39] who used the CFIR in the implementation of an on-line frailty tool in PHC. High complexity was due to the multiple program components and the need for changes in practice routines, and integrating the tool into the EMR was recommended to improve accessibility [39]. Use of the EMR is an important tool for interdisciplinary PHC [40, 41], especially in rural settings where team members are not co-located, but it was not without challenges in the current study.

Within **inner setting**, establishing and maintaining *leadership engagement* was essential to approving and encouraging the intervention. Leadership and managerial support were top facilitators in a review of implementation research in dementia care [10] and are often linked to relative priority and resources [29]. *Tension for change* motivated initial and ongoing participation in the study, although physicians were less likely to have concerns about current practices. Sopcak et al. [31] described a “disconnect” between perceptions of physicians and other providers regarding the need for implementation of a chronic disease prevention program in PHC settings. Similarly, Boise et al. [42] found that physician response to a dementia screening intervention in rural PHC settings was mixed, compared to medical assistants, who perceived the intervention positively. *Compatibility* with existing work patterns was a challenge in the current study, as new ways of working as a team were required. Sopcak et al. [31] used the CFIR framework to study implementation of a chronic disease intervention in PHC and noted that when new approaches impact the routine and workflow, and requires people to work in new ways, regular communication is needed to resolve issues and move the implementation forward. In the current study it was challenging to organize frequent face-to-face meetings due to busy clinical schedules, lack of co-location of the team, and distance of the researchers from the team. Over the study period the researchers travelled 13,780 kms to meet with the PHC team in their community.

Compatibility interacted with *resources* in that the clinic model required more team member time than usual care. A review of implementation barriers and facilitators of evidence-based dementia care found workload and time constraints as a dominant theme [10]. Our findings are consistent with Boise et al. [42] who implemented a protocol for dementia diagnosis and screening in rural PHC settings who found that understanding the routines, available resources, and attitudes in rural settings was essential to developing interventions that were acceptable in real-world PHC contexts. Boise et al. [42] attributed the modest uptake of the intervention by physicians, despite satisfaction with the training and increased confidence, to the challenges of practice change in busy clinical environments and perceptions that the intervention was not a priority. Warner et al. [39] found that opportunity costs (completing a frailty assessment in PHC vs. seeing another patient) were greater for physicians than nurse practitioners, whose practice was more flexible and compatible with the intervention. This difference in practice patterns may have contributed to the greater involvement of the nurse practitioner in the current study.

The **process** domain was also important. Even with the support of leaders and *champions*, the lack of a formally appointed internal implementation leader was a barrier. Team members could not add this role to their regular responsibilities, and there was no one on-site with the designated authority, credibility, and capacity to facilitate implementation. Facilitation by the researchers (*external change agents)* was therefore important to maintaining momentum, but the remote location made it difficult to have a sustained physical presence. Although Boise et al. [42] recommended on-site assistance from research staff in implementing a dementia screening protocol in rural PHC teams, this was not feasible in the current study given the intermittent nature of the one-day clinics and the cost and logistics of having research staff relocate or travel frequently to the community.

The absence of sustainability as a construct has been identified as a gap in the CFIR framework [32]. Participants in the current study identified the importance of ongoing researcher facilitation in sustainability, and time factors and turnover of team members as key barriers. Turnover resulted in loss of capacity that had been developed, and engaging and orienting new team members was challenging because of existing workloads and responsibilities. When stakeholders are engaged early in the process they may be more invested in sustaining it [29, 31], thus new team members may lack this commitment.

### Impact of rural context

The lack of co-location of PHC team members made it more difficult and time-consuming for them to meet and limited the opportunities for informal conversations, which is key to interprofessional collaboration [40, 43]. A study of high vs. low performing PHC teams [41] found that co-location supported team development by facilitating hallway conversations and learning about each others’ roles. The five-hour drive from the researchers’ university to the rural community of the PHC team, and hazards of travel in winter, also limited the number of face-to-face meetings. Collaborative community-based participatory research methods have been identified as a useful approach for addressing the needs of underserved rural and remote locations [44]. However, as Ritchie et al. [45] have reported, the principles of this approach are more difficult to apply when communities are not in close proximity to the researchers, as geographic distance limits the frequency of face-to-face interaction that is important for building relationships. Physician recruitment and retention had an impact in the current study, and is an ongoing challenge in rural communities in Canada and globally [46, 47]. Rural and remote PHC teams are more affected by workforce turnover and availability than urban teams [41].

### Study strengths and limitations

The study provides an in-depth examination of the implementation process, but further research is needed to understand how these findings might apply in other settings. This research focused on PHC teams because of provincial policy directions. The factors influencing implementation and feasibility in fee-for-service settings may be very different. The study was affected by limitations of the rural setting as noted above, particularly workforce turnover, recruitment, and retention of PHC team members. At the end of the study, only two of the original team members were still with the team. Heavy workloads exacerbated by vacancies in PHC team positions affected team members’ availability to participate in some components of the study, including the interviews. Lack of direct patient and family input is also a limitation, and an identified gap in the CFIR [32]. We are currently exploring patient and family perspectives of the intervention. It is recommended that dementia researchers take sex and/or gender differences into account in study design and reporting [48]. Sex and gender differences were not explicitly explored in this study as it focused on stakeholders’ perceptions of the intervention development and implementation process, but will be explored as the intervention is sustained and expanded, and we have sufficient patient and family numbers to measure outcomes associated with the PHC memory clinic model. Strengths of this research include the in-depth and longitudinal perspective, inclusion of multiple stakeholders, and use of a guiding theoretical framework to capture the complexity of implementation in a rural context. As noted by others [29] the strength of the CFIR is its comprehensiveness, but it does create complexity in applying the coding framework and determining the relative importance of multiple constructs. Because of its utility in identifying factors influencing implementation, we are continuing to use the CFIR to monitor long-term sustainability of the intervention. The lessons learned from the process evaluation with the first team have been applied as we expand the model to additional teams in the region.

## Conclusion

This study reinforces the “problem solving” view of rural communities as change agents and innovators [49]. The RaDAR team’s 20-year experience in rural dementia research, and evidenced in the current study, is that rural health care providers and those involved in planning services are resourceful, collaborative, engaged with their community, and innovative in addressing community needs. The PHC team’s commitment to the long iterative process of co-designing and implementing the rural PHC intervention indicates their commitment to improving practice gaps for people with dementia and their families, despite the many challenges. The current study identified key barriers and facilitators in development and implementation of a best practice model of rural PHC for dementia than can inform future PHC interventions for dementia in rural and remote contexts. The key CFIR domains that emerged over this longitudinal study were the inner setting of the PHC team, the intervention itself, and the engagement of key individuals in the implementation process. The CFIR provided a structure for understanding the many influences at play when implementing a complex intervention such as the memory clinic into an equally complex PHC setting. Study findings indicate that even within rural settings that typically have fewer resources to draw on, evidence-based interventions can be successfully developed and implemented. The researcher-academic partnership and use of an implementation framework were important to this outcome. Continued use of the CFIR to monitor sustainability of the intervention in the initial PHC team and scaling up to other teams will expand our understanding of factors influencing the maintenance and spread of an intervention aimed at addressing identified gaps in dementia care in rural PHC settings.

## Supplementary information

**Additional file 1.** Interview guide: Pre-implementation focus group. This interview guide was developed from the Consolidated Framework for Implementation Research (CFIR) constructs and definitions of Damschroder et al., 2009 [26]. Color coding was used to show priority of question order within each domain, to ensure questions identified as most critical at the pre-implementation phase were asked in the time available.

**Additional file 2.** Interview guide: Post-implementation telephone interviews. This interview guide was developed from the CFIR constructs and definitions of Damschroder et al., 2009 [26] and the interview guide tools that became available on the CFIR website at the post-implementation phase of the study. See https://cfirguide.org/ and http://cfirwiki.net/guide/app/index.html#/guide_select. Given the brief telephone interview format, selected questions from each domain were included based on findings of the process evaluation to that point.

**Additional file 3.** Operational definitions for CFIR domains and constructs. This file provides definitions of the CFIR domains and constructs (adapted from Damschroder et al. [26]) that were operationalized in the context of the current study.

## Data Availability

The datasets generated and analyzed during the current study are not publically available for reasons of participant confidentiality due to small sample size.

## Abbreviations

CFIR: Consolidated Framework for Implementation Research
EMR: Electronic medical record
PC-DATATM: Primary Care: Dementia Assessment & Treatment Algorithm
PHC: Primary health care
RaDAR: Rural Dementia Action Research Team
