# Supplementary material for: Barriers and facilitators to development and implementation of a rural primary health care intervention for dementia: a process evaluation
Source: BMC Health Serv Res. 2019 Oct 17;19:709. doi: 10.1186/s12913-019-4548-5 (PMC6798332; doi:10.1186/s12913-019-4548-5)
Supplement: Supplementary file 1 — Additional file 1. Interview guide: Pre-implementation focus group. This interview guide was developed from the Consolidated Framework for Implementation Research (CFIR) constructs and definitions of Damschroder et al., 2009 [26]. Color coding was used to show priority of question order within each domain, to ensure questions identified as most critical at the pre-implementation phase were asked in the time available. [file 12913_2019_4548_MOESM1_ESM.docx]

**ADDITIONAL FILE 1: Pre-implementation Focus Group Guide**

**Demographics and professional roles of focus group participants**

Current job title

Current job responsibilities

Birth year

Gender

**Question order**

First priority

Second priority

Third/last priority

Third/last priority

| **Pre-Implementation with PHC Team #1**  **Focus Group Guiding Questions**  (Constructs and definitions from the *Consolidated Framework for Implementation Research*, Damshroder et al. 2009) | | |
| --- | --- | --- |
| **Construct** | **Construct definition** | **Guiding Question** |
| **I. INNOVATION CHARACTERISTICS** | | |
| **Innovation characteristics**  Intervention Source | Perception of key stakeholders about whether the intervention is externally or internally developed. | How did you first become aware of the RaDAR project?  What was your first reaction when you heard about the project (e.g., did/did not want to learn more or did not have an immediate reaction)? |
| **Innovation characteristics**  Evidence Strength & Quality | Stakeholders’ perceptions of the quality and validity of evidence supporting the belief that the intervention will have desired outcomes. | Have you heard about the RaDAR project from other individuals in your health region (e.g., health region administrators, others) or from other sources (e.g., internet, newsletter, other electronic/printed source)?  Probe. Who or what were these sources?  Given what you heard, did you believe that the RaDAR project would (or would not) be effective? |
| **Innovation characteristics**  Relative advantage | Stakeholders’ perception of the advantage of implementing the intervention versus an alternative solution. | Other than the RaDAR project, are you aware of any other similar projects in your health region, or in other health regions, that are focused on quality improvement in dementia care?  Probe. How does the RaDAR project compare to these other programs/projects?  **Link to other CDM projects.** Thinking of quality improvement projects related to other chronic diseases that your team has been involved in (e.g., CDM-QIP diabetes, COPD, CAD, heart failure), what have been the benefits of these projects to date (for patients and personally)? |
| **Innovation characteristics**  Adaptability | The degree to which an intervention can be adapted, tailored, refined, or reinvented to meet local needs. | Given what you know the RaDAR project, do you think that the core intervention can be tailored to fit your team’s needs? |
| **Innovation characteristics**  Trialability | The ability to test the intervention on a small scale in the organization, and to be able to reverse course (undo implementation) if warranted. | n/a (overlap with ‘complexity) |
| **Innovation characteristics**  Complexity | Perceived difficulty of implementation, reflected by duration, scope, radicalness, disruptiveness, centrality, and intricacy and number of steps required to implement | How difficult do you think it will be to implement the RaDAR project within your team (e.g., on a scale of 1 to 10)?  What do you think might be the most and least challenging aspects of implementing the RaDAR project within your team (e.g., team communication, IT support, willingness to learn, etc.)? |
| **Innovation characteristics**  Design Quality and Packaging | Perceived excellence in how the intervention is bundled, presented, and assembled | n/a at this stage |
| **Innovation characteristics**  Cost | Costs of the intervention and costs associated with implementing that intervention including investment, supply, and opportunity costs. | n/a (will derive from PHC facilitator and other info sources) |
| **II. OUTER SETTING** | | |
| **Outer Setting**  Patient Needs & Resources | The extent to which patient needs, as well as barriers and facilitators to meet those needs are accurately known and prioritized by the organization. | Thinking about your patients living with dementia and their families, what would you say are some of their most pressing needs?  In your team, what might be some of the highest priorities (i.e., largest gaps) in terms of improving the care that you provide individuals with dementia? |
| **Outer Setting**  Cosmopolitanism | The degree to which an organization is networked with other external organizations. | n/a (will derive from PHC facilitator and other info sources) |
| **Outer Setting**  Peer Pressure | Mimetic or competitive pressure to implement an intervention; typically because most or other key peer or competing organizations have already implemented or in a bid for a competitive edge | Out of the 8 PHC teams in your health region, what was it about your team that made it the first to work on the RaDAR project? |
| **Outer Setting**  External Policy & Incentives | A broad construct that includes external strategies to spread interventions including policy and regulations (governmental or other central entity), external mandates, recommendations and guidelines, pay-for-performance, collaboratives, and public or benchmark reporting. | Do members of your team receive incentives - that come specifically from the Ministry of Health or your provincial professional organization - to take part in QI projects related to other chronic diseases, (e.g., CDM-QIP diabetes, COPD, CAD, heart failure)? Are these incentives effective in terms of implementing the QI strategies?  Can you think of any past or present policies or initiatives – at the level of your health region or Ministry of Health – that have paved the way for the RaDAR project to be tested? |
| **III. INNER SETTING** | | |
| **Inner Setting**  Structural Characteristics | The social architecture, age, maturity, and size of an organization. | n/a (will derive from PHC facilitator and other info sources) |
| **Inner Setting**  Networks & Communications | The nature and quality of webs of social networks and the nature and quality of formal and informal communications within an organization. | What forms of communication within the team have been successful in terms of care for patients with other chronic diseases (e.g., in-person, telephone, email, EMR, other)?  How is care typically coordinated for patients with other chronic diseases among the clinical members of your team (i.e., who performs assessment, diagnosis, and ongoing management)?  Are there ways that care coordination within the team could be improved, when it comes to patient care for individuals with dementia?  What might be some barriers – and existing strengths - to improving care coordination among the clinical members of your team, for patients with dementia? |
| **Inner Setting**  Culture | Norms, values, and basic assumptions of a given organization. | What are some words/terms that you would use to describe the organizational culture of your team?  How do you think the organizational culture of your team will affect your work on the RaDAR project? |
| **Inner Setting**  Implementation Climate | The absorptive capacity for change, shared receptivity of involved individuals to an intervention and the extent to which use of that intervention will be rewarded, supported, and expected within their organization. | See #a-f below |
| **Inner Setting**  **- Implementation climate**  a. Tension for Change | The degree to which stakeholders perceive the current situation as intolerable or needing change. | What prompted your team to agree to be the first in the health region to work on the RaDAR project?  Probe. What factors were considered when your team made this decision? |
| **Inner Setting**  **- Implementation climate**  b. Compatibility | The degree of tangible fit between meaning and values attached to the intervention by involved individuals, how those align with individuals’ own norms, values, and perceived risks and needs, and how the intervention fits with existing workflows and systems. | n/a (difficult to assess each individual’s norms, values, etc. within a focus group) |
| **Inner Setting**  **- Implementation climate**  c. Relative Priority | Individuals’ shared perception of the importance of the implementation within the organization. | Thinking of the time that will be spent on the RaDAR project, what might be some competing priorities that would make it difficult to devote time to the RaDAR project?  Probe. Are these short-term or ongoing competing priorities? |
| **Inner Setting**  **- Implementation climate**  d. Organizational Incentives & Rewards | Extrinsic incentives such as goal-sharing awards, performance reviews, promotions, and raises in salary and less tangible incentives such as increased stature or respect. | How will your team benefit by participating in the RaDAR project?  Do members of your team receive incentives - that come specifically from within your team or within your health region - to take part in QI projects related to other chronic diseases? Are these incentives effective in terms of implementing the QI strategies? |
| **Inner Setting**  **- Implementation climate**  e. Goals and Feedback | The degree to which goals are clearly communicated, acted upon, and fed back to staff and alignment of that feedback with goals. | Has your team had a chance yet to define particular goals associated with the RaDAR project, and site?  Probe. What have you learned from other QI projects about defining and targeting goals that could be applied to the RaDAR project (e.g., related to other chronic diseases)? |
| **Inner Setting**  **- Implementation climate**  f. Learning Climate | A climate in which: a) leaders express their own fallibility and need for team members’ assistance and input; b) team members feel that they are essential, valued, and knowledgeable partners in the change process; c) individuals feel psychologically safe to try new methods; and d) there is sufficient time and space for reflective thinking and evaluation. | How would you describe your team’s environment for trying new methods of patient care for individuals with dementia, at this point in time – do you feel encouraged to change practice?  Probe. What might be some challenges within your team, to testing the RaDAR project at this time? |
| **Inner Setting**  Readiness for Implementation | Tangible and immediate indicators of organizational commitment to its decision to implement an intervention. | See #a-c below |
| **Inner Setting**  **- Readiness for implementation**  a. Leadership Engagement | Commitment, involvement, and accountability of leaders and managers with the implementation. | How would you characterize the level of support from your health region, for your team’s involvement in the RaDAR project?  Probe. Can you give some specific examples of support (e.g., extra staff, devoted time from existing staff, extra space, more funding, etc.)? |
| **Inner Setting**  **- Readiness for implementation**  b. Available Resources | The level of resources dedicated for implementation and on-going operations including money, training, education, physical space, and time. | Probe. What resources do you have in place for patients with other chronic diseases, that could be drawn upon for the purposes of the RaDAR project? Discuss how each of the following resources contributes to diagnosis and management for individuals with chronic diseases:  - multidisciplinary team  - ongoing care management  - regular patient follow-up  - formal training, interactive case-based experiential learning, access to specialists  - standard tools, protocols, guidelines  - access to IT resources (e.g., EMR)  - education/support to individuals and caregivers  - other? |
| **Inner Setting**  **- Readiness for implementation**  c. Access to knowledge and information | Ease of access to digestible information and knowledge about the intervention and how to incorporate it into work tasks. | Given your team’s involvement in other QI projects related to chronic diseases, how do you access information on the effectiveness of these projects/interventions for your practice? |
| **IV. CHARACTERISTICS OF INDIVIDUALS** | | |
| **Characteristics of individuals**  Knowledge & Beliefs about the Intervention | Individuals’ attitudes toward and value placed on the intervention as well as familiarity with facts, truths, and principles related to the intervention. | On a scale of 1 to 10, with 10 being highest, how necessary is the RaDAR project to your patients’ needs?  What do you think we as researchers need to keep in mind for the RaDAR project to be successful within your team? |
| **Characteristics of individuals**  Self-efficacy | Individual belief in their own capabilities to execute courses of action to achieve implementation goals. | What do you see as your particular role, within the RaDAR project? |
| **Characteristics of individuals**  Individual Stage of Change | Characterization of the phase an individual is in, as he or she progresses toward skilled, enthusiastic, and sustained use of the intervention. | On a scale of 1 to 10, with 10 being highest, how would you describe your team’s interest in participating in the RaDAR project? |
| **Characteristics of individuals**  Individual Identification with Organization | A broad construct related to how individuals perceive the organization and their relationship and degree of commitment with that organization. | n/a (difficult to assess within a focus group) |
| **Characteristics of individuals**  Other Personal Attributes | A broad construct to include other personal traits such as tolerance of ambiguity, intellectual ability, motivation, values, competence, capacity, and learning style. | n/a (difficult to assess within a focus group) |
| **V. PROCESS** | | |
| **Process**  Planning | The degree to which a scheme or method of behavior and tasks for implementing an intervention are developed in advance and the quality of those schemes or methods. | Have any plans been put into motion, at the team level or health region level, to track RaDAR project outcomes?  What plans need to be made to put the RaDAR project into motion within the team? |
| **Process**  Engaging | Attracting and involving appropriate individuals in the implementation and use of the intervention through a combined strategy of social marketing, education, role modeling, training, and other similar activities. | See #a-d below |
| **Process**  **Engaging**  a. Opinion Leaders | Individuals in an organization who have formal or informal influence on the attitudes and beliefs of their colleagues with respect to implementing the intervention | Other than the current members of the team, and those outside the team that are currently involved, are there any particular opinion leaders or champions have been involved in the RaDAR project? Are there any particular opinion leaders or champions that should be involved in the RaDAR project as we move forward?  Probe. Thinking of your involvement in other QI initiatives and in improving care for individuals with other chronic diseases, have there been particular individuals who stood out as opinion leaders or champions? |
| **Process**  **Engaging**  b. Formally appointed internal implementation leaders | Individuals from within the organization who have been formally appointed with responsibility for implementing an intervention as coordinator, project manager, team leader, or other similar role. | Have any team members been given formal responsibility for implementing the RaDAR project?  *Probe*. Who are these individuals and what are their roles within the project? |
| **Process**  **Engaging**  c. Champions | “Individuals who dedicate themselves to supporting, marketing, and ‘driving through’ an [implementation]” overcoming indifference or resistance that the intervention may provoke in an organization. | See #a above |
| **Process**  **Engaging**  d. External Change Agents | Individuals who are affiliated with an outside entity who formally influence or facilitate intervention decisions in a desirable direction. | See #a above |
| **Process**  Executing | Carrying out or accomplishing the implementation according to plan. | n/a until the implementation stage |
| **Process**  Reflecting & Evaluating | Quantitative and qualitative feedback about the progress and quality of implementation accompanied with regular personal and team debriefing about progress and experience. | Have any processes been put into place to gather information on the progress of the RaDAR project, at the team level or health region level? |
| **Process**  Scale-up, sustainability, and spread |  | Are you aware of any processes that have been put into place to sustain the project within this team and scale-up the project to other teams within the health region? |
