# Supplementary material for: Barriers and facilitators to development and implementation of a rural primary health care intervention for dementia: a process evaluation
Source: BMC Health Serv Res. 2019 Oct 17;19:709. doi: 10.1186/s12913-019-4548-5 (PMC6798332; doi:10.1186/s12913-019-4548-5)
Supplement: Supplementary file 2 — Additional file 2. Interview guide: Post-implementation telephone interviews. This interview guide was developed from the CFIR constructs and definitions of Damschroder et al., 2009 [26] and the interview guide tools that became available on the CFIR website at the post-implementation phase of the study. See https://cfirguide.org/ and http://cfirwiki.net/guide/app/index.html#/guide_select. Given the brief telephone interview format, selected questions from each domain were included based on findings of the process evaluation to that point. [file 12913_2019_4548_MOESM2_ESM.doc]

**ADDITIONAL FILE 2: Post-implementation Telephone Interview Guide**

**Consent form**

- Ask the participant if they have had an opportunity to read the consent form.
  - If yes, ask the participant if they consent to participate in the interview, and then continue on to the preamble.
  - If no, either email the form or read aloud before asking for verbal consent and continuing on.

**Advise the participant that the interview is being audio-recorded**

**Preamble to be read aloud**

- The main purpose of this interview is to evaluate the contextual factors that influence the implementation and sustainability of the RaDAR intervention for dementia care in primary health care teams.
- The interview is organized in 4 sections. The first section collects information about your demographics and professional role. The last three sections focus on the pre-implementation stage of the project, then the implementation stage, and finally the post-implementation stage.
- Throughout the interview, I will use the word “team”. When I use this word, I am referring specifically to the health care providers and staff that work together in the (insert name of town) primary health care clinic.
- When I use the term, “RaDAR project” I am referring to all of the materials, communications, and time spent working directly or indirectly to develop and implement the RaDAR intervention.
- And the term “RaDAR intervention” refers to the team processes related to dementia care that were developed during this project, the education sessions provided by Drs. Kirk and Seitz, the adapted PC-DATA flow sheet currently in the Med Access EMR, and the RaDAR handbook that includes the work standards, scripts, and other materials.

**Demographics and professional roles**

What is your current job title(s)?

What is your birth year?

And what is your gender (you may choose not to ask).

Do you collaborate with other health care providers in the (insert name of town) team? Can you explain the nature of this collaboration and how this it might differ depending on the provider, if at all?

(Inner Setting – Networks & Communications)

How often do you interact face-to-face with other members of the (insert name of town) team (e.g., daily, weekly, less often)?

(Inner Setting – Networks & Communications)

How often do you have contact with other members of the (insert name of town) team, other than face to face, such as by telephone, through electronic medical record, email, or otherwise (e.g., daily, weekly, less often)?

(Inner Setting – Networks & Communications)

**Pre-Implementation**

**If you can think back to the time that the RaDAR project first began in your team:**

1. Were there any particular areas of dementia care that you viewed as needing the most change, in your team (e.g., diagnosis, management, driving, other)?

*Probe*. What led you to believe that?

(Implementation Climate – Tension for Change)

1. Did you believe that the RaDAR project might change dementia care in your team?

If so, how?

*Probe*. Please explain, e.g., how did you think the RaDAR project would (or would not) improve dementia care at the time?

(Characteristics of Individuals – Knowledge and beliefs about the intervention)

1. What did you know about the RaDAR project before becoming involved (e.g., what did you know about the project goals, what were you expected to contribute)?

*Probe.* Where did this evidence come from? E.g., colleagues, supervisor/manager, other?

(Intervention Characteristics - Evidence Strength and Quality)

1. Do you think you knew enough about the RaDAR intervention before becoming involved?

*Probe*. If no, what additional information would you like to have?

(Intervention Characteristics - Evidence Strength and Quality)

1. Did you think you would be able to contribute to developing the RaDAR intervention?

*Probe*. If no, what additional skills/training/other resources would you liked to have had?

(Characteristics of Individuals – Self-efficacy)

1. When you became involved in the RaDAR project, was your team also trying to meet other priorities aside from dementia care?

If so, how did this affect your team’s ability or capacity to take part in the RaDAR project?

*Probe*. What were these initiatives?

(Inner Setting – Implementation climate – Relative priority)

1. Did your PHC team discuss the possible benefits and challenges - specifically to your team and/or patients – that might result from involvement in the RaDAR project?

*Probe*. What were those benefits and challenges?

(Intervention Characteristics – Relative advantage)

**Implementation**

**Thinking now about the steps involved in the development and implementation phase of the RaDAR project in your team:**

“Champions” have been defined as “individuals who dedicate themselves to supporting, marketing, and ‘driving through’ an [implementation], overcoming indifference or resistance that the intervention may provoke in an organization” (Damschroder et al. 2009).

Outside of your team, was there anyone you would call a “champion” of the RaDAR project?

*Probe.* What do you think motivated these individuals to be champions of the RaDAR project?

(Process – Engaging – Opinion leaders)

Within your team, were there any individual(s) in particular who took on most of the responsibility for the RaDAR project (e.g., working with the RaDAR team, helping to develop the intervention, implementing the intervention, testing the intervention)?

*Probe.* What specific responsibilities did the individual(s) take on?

(Process – Engaging – Champions)

Would you say that there were sufficient resources available to develop and implement the RaDAR intervention (e.g., time, physical space, funding, training, and information technology)?

*Probe*. How were the resources sufficient or insufficient?

*Probe*. Did your team receive extra personnel, information technology, funding, or other incentives?

(Inner setting – Readiness for Implementation – Available resources)

Did **your team** encounter any barriers to developing and implementing the RaDAR intervention?

*Probe*. Barriers related to professional scope of practice?

*Probe*. Barriers related to availability of staff or resources?

*Probe*. Barriers related to team communication?

*Probe*. Information technology barriers?

*Probe*. Barriers related staff, or patient/caregiver resistance?

(Inner setting – Readiness for Implementation)

Did **you** specifically encounter any barriers to embedding the RaDAR intervention **within your regular practice?**

*Probe*. Barriers related to professional scope of practice?

*Probe*. Barriers related to availability of staff or resources?

*Probe*. Barriers related to team communication?

*Probe*. Information technology barriers?

*Probe*. Barriers related staff, or patient/caregiver resistance?

(Inner setting – Readiness for Implementation)

Were there any individuals, or processes, or infrastructure, that facilitated the RaDAR intervention?

*Probe.* At the health region level?

*Probe.* At the team level?

*Probe.* Personal or professional facilitators?

*Probe.* Other?

(Inner Setting)

**Post-Implementation**

**Now that the RaDAR intervention has been developed and implemented in your team:**

1. To what degree would you say that the RaDAR intervention has influenced dementia care within your PHC team?

*Probe*. No effect, minor affect, neutral, moderate affect, major affect?

(Process - Executing)

How has the RaDAR intervention influenced the way you practice with respect to dementia care, if at all?

*Probe*. Please explain.

(Process - Executing)

1. Did the RaDAR intervention address the area(s) of dementia care that you thought should be addressed?

*Probe*. How so?

*Probe*. Are there other areas that haven’t been addressed, that should be addressed?

(Process- Reflecting and Evaluating)

Does your team regularly meet to discuss any practices or outcomes associated with the RaDAR intervention?

*Probe*. Please explain.

(Process - Executing)

How embedded has the RaDAR intervention become in the ongoing work of the PHC team?

*Probe*. Has the PHC team sustained any or all of practices associated with the RaDAR project?

(Process - Executing)

Are there any changes that you would like to suggest be made to the RaDAR intervention at this time –for example team processes, education sessions, or modifications/additions to the EMR flow sheet or materials in the RaDAR handbook?

*Probe*. Explore.

(Process- Reflecting and Evaluating)

What actions do you think should be taken – by RaDAR or your team - to help sustain the RaDAR intervention in your team?

(Process- Reflecting and Evaluating)

What actions should your supervisors take, and others outside your team, to help sustain the RaDAR intervention in your team?

(Process- Reflecting and Evaluating)

What advice do you have for other teams currently implementing, or thinking of implementing, the RaDAR intervention?

(Process – Reflecting and Evaluating)
