# Supplementary material for: Barriers and facilitators to development and implementation of a rural primary health care intervention for dementia: a process evaluation
Source: BMC Health Serv Res. 2019 Oct 17;19:709. doi: 10.1186/s12913-019-4548-5 (PMC6798332; doi:10.1186/s12913-019-4548-5)
Supplement: Supplementary file 3 — Additional file 3. Operational definitions for CFIR domains and constructs. This file provides definitions of the CFIR domains and constructs (adapted from Damschroder et al. [26]) that were operationalized in the context of the current study. [file 12913_2019_4548_MOESM3_ESM.docx]

Additional File 3. Operational Definitions for CFIR domains and constructs used in this study

| **Domain and construct** | **Definition and inclusion criteria** |
| --- | --- |
| 1. **Innovation Characteristics** |  |
| 1. Innovation Source | Definition: Perception of key stakeholders about whether the Rural PHC Model for Dementia is externally or internally developed.  Inclusion Criteria: Include statements about the source of the Rural PHC Model for Dementia and the extent to which interviewees view the change as internal to Team 1. |
| 1. Evidence Strength & Quality | Definition: Stakeholders’ perceptions of the quality and validity of evidence supporting the belief that the Rural PHC Model for Dementia will have desired outcomes.  Inclusion Criteria: Include statements regarding awareness of evidence and the strength and quality of evidence, as well as the absence of evidence or a desire for different types of evidence, such as pilot results instead of evidence from the literature. |
| 1. Relative Advantage | Definition: Stakeholders’ perception of the advantage of implementing the Rural PHC Model for Dementia versus an alternative solution or the status quo.  Inclusion Criteria: Include statements that demonstrate the Rural PHC Model for Dementia is better (or worse) than existing programs or the status quo. |
| 1. Adaptability | Definition: The degree to which a Rural PHC Model for Dementia can be adapted, tailored, refined, or reinvented to meet local needs.  Inclusion Criteria: Include statements regarding the (in)ability to adapt the Rural PHC Model for Dementia to their context, e.g., complaints about the rigidity of the protocol. |
| 1. Trialability | Definition: The ability to test the Rural PHC Model for Dementia on a small scale in Team 1, and to be able to reverse course (undo implementation) if warranted.  Inclusion Criteria: Include statements related to whether the site piloted the Rural PHC Model for Dementia in the past or has plans to in the future, and comments about whether they believe it is (im)possible to conduct a pilot. |
| 1. Complexity | Definition: Perceived difficulty of implementing the Rural PHC Model for Dementia, reflected by duration, scope, radicalness, disruptiveness, centrality, and intricacy and number of steps required to implement.  Inclusion Criteria: Code statements regarding the complexity of the innovation itself.  Exclusion Criteria: Exclude statements regarding the complexity of implementation. |
| 1. Design Quality & Packaging | Definition: Perceived excellence in how the Rural PHC Model for Dementia is bundled, presented, and assembled.  Inclusion Criteria: Include statements regarding the quality of materials and packaging. |
| 1. Cost | Definition: Costs of the Rural PHC Model for Dementia and costs associated with implementing the Rural PHC Model for Dementia.  Inclusion Criteria: Include statements related to the cost of the Rural PHC Model for Dementia and its implementation. |
| 1. **Outer Setting** |  |
| 1. Needs & Resources of Those Served by the Organization | Definition: The extent to which the needs of those served by Team 1 (e.g., patients and families), as well as barriers and facilitators to meet those needs, are accurately known and prioritized by the Team 1.  Inclusion Criteria: Include statements demonstrating (lack of) awareness of the needs and resources of those served by Team 1:   - - perceived need for the Rural PHC Model for Dementia based on the needs of those served by Team 1 and if the Rural PHC Model for Dementia will meet those needs.   - barriers and facilitators of those served by Team 1 to participating in the Rural PHC Model for Dementia.   - whether or not awareness of the needs and resources of those served by Team 1 influenced the implementation or adaptation of the Rural PHC Model for Dementia. |
| 1. Cosmopolitanism | Definition: The degree to which Team 1 is networked with other external organizations.  Inclusion Criteria: Include descriptions of outside group memberships and networking done outside Team 1. |
| 1. Peer Pressure | Definition: Mimetic or competitive pressure to implement the Rural PHC Model for Dementia, typically because most or other key peer or competing organizations have already implemented or are in a bid for a competitive edge.  Inclusion Criteria: Include statements about perceived pressure or motivation from other entities or organizations in the local geographic area or system to implement the Rural PHC Model for Dementia. |
| 1. External Policy & Incentives | Definition: External strategies to spread the Rural PHC Model for Dementia including policy, regulations, recommendations, guidelines, collaboratives, benchmark reporting.  Inclusion Criteria: Include descriptions of external performance measures. |
| 1. **Inner Setting** |  |
| 1. Structural Characteristics | Definition: The social architecture, age, maturity, and size of Team 1. |
| 1. Networks & Communications | Definition: The nature and quality of webs of social networks, and the nature and quality of formal and informal communications within Team 1.  Inclusion Criteria: Include statements about networking, communication, and relationships in Team 1, such as descriptions of meetings or other methods to keep people connected and informed, and statements about team formation, quality, functioning. |
| 1. Culture | Definition: Norms, values, and basic assumptions of a given Team 1. |
| 1. Implementation Climate |  |
| 1. Tension for Change | Definition: The degree to which stakeholders perceive the current situation as intolerable or needing change.  Inclusion Criteria: Include statements that (do not) demonstrate a strong need for the Rural PHC Model for Dementia and/or that the current situation is untenable, e.g., statements that the Rural PHC Model for Dementia is absolutely necessary or that the Rural PHC Model for Dementia is redundant with other programs. |
| 1. Compatibility | Definition: How the Rural PHC Model for Dementia fits with existing workflows and systems.  Inclusion Criteria: Include statements that demonstrate the level of compatibility the Rural PHC Model for Dementia has with organizational values and work processes (of Team 1). |
| 1. Relative Priority | Definition: Individuals’ shared perception of the importance of the implementation within Team 1.  Inclusion Criteria: Include statements that reflect the relative priority of the Rural PHC Model for Dementia, e.g., statements related to change fatigue in Team 1 due to implementation of many other programs. |
| 1. Organizational Incentives & Rewards | Definition: Extrinsic incentives such as awards, performance reviews, promotions, and raises in salary, and less tangible incentives such as increased stature or respect.  Inclusion Criteria: Include statements related to whether organizational incentive systems are in place (in Team 1) to foster (or hinder) implementation, e.g., rewards or disincentives for staff engaging in the Rural PHC Model for Dementia. |
| 1. Goals & Feedback | Definition: The degree to which goals are clearly communicated, acted upon, and fed back to staff, and alignment of that feedback with goals.  Inclusion Criteria: Include statements related to the (lack of) alignment of implementation and Rural PHC Model for Dementia goals with larger organizational goals (of Team 1), as well as feedback to staff regarding those goals, e.g., regular audit and feedback showing any gaps between the current organizational status and the goal. |
| 1. Learning Climate | Definition: A climate in which: 1. Leaders express their own fallibility and need for team members’ assistance and input; 2. Team members feel that they are essential, valued, and knowledgeable partners in the change process; 3. Individuals feel psychologically safe to try new methods; and 4. There is sufficient time and space for reflection and evaluation.  Inclusion Criteria: Include statements that support (or refute) the degree to which key components of Team 1 exhibit a “learning climate.” |
| 1. Readiness for Implementation |  |
| 1. Leadership Engagement | Definition: Commitment, involvement, and accountability of leaders and managers with the implementation of the Rural PHC Model for Dementia.  Inclusion Criteria: Include statements regarding the level of engagement of organizational leadership (within and/or outside of Team 1). |
| 1. Available Resources | Definition: The level of resources dedicated for implementation and on-going operations including physical space and time (within and outside Team 1).  Inclusion Criteria: Include statements related to the presence or absence of resources specific to the Rural PHC Model for Dementia that is being implemented. |
| 1. Access to Knowledge & Information | Definition: Ease of access to digestible information and knowledge about the Rural PHC Model for Dementia and how to incorporate it into work tasks.  Inclusion Criteria: Include statements related to implementation leaders' and users' access to knowledge and information regarding use of the program, i.e., training on the mechanics of the program. |
| 1. **Characteristics of Individuals** |  |
| 1. Knowledge & Beliefs about the Innovation | Definition: Individuals’ attitudes toward and value placed on the Rural PHC Model for Dementia, as well as familiarity with facts, truths, and principles related to the Rural PHC Model for Dementia. |
| 1. Self-efficacy | Definition: Individual belief in their own capabilities to execute courses of action to achieve implementation goals. |
| 1. Individual Stage of Change | Definition: Characterization of the phase an individual is in, as s/he progresses toward skilled, enthusiastic, and sustained use of the Rural PHC Model for Dementia. |
| 1. Individual Identification with Organization | Definition: A broad construct related to how individuals perceive Team 1, and their relationship and degree of commitment with Team 1. |
| 1. Other Personal Attributes | Definition: A broad construct to include other personal traits such as tolerance of ambiguity, intellectual ability, motivation, values, competence, capacity, learning style. |
| 1. **Process** |  |
| 1. Planning | Definition: The degree to which a scheme or method of behavior and tasks for implementing the Rural PHC Model for Dementia are developed in advance, and the quality of those schemes or methods. |
| 1. Engaging | Definitions of the constructs in this domain were expanded to include not just engaging, but also retaining appropriate individuals in the implementation and use of the Rural PHC Model, and to include all activities of these individuals related to implementation. |
| 1. Opinion Leaders | Definition: Individuals outside of Team 1 but within the health region that have formal or informal influence on the attitudes and beliefs of their colleagues with respect to implementing the Rural PHC Model for Dementia.  Inclusion Criteria: Include statements related to engagement strategies and outcomes, e.g., how the opinion leader became engaged with the Rural PHC Model for Dementia and what their role is in implementation. |
| 1. Formally Appointed Internal Implementation Leaders | Definition: Individuals within or outside Team 1 who have been formally appointed with responsibility for implementing a Rural PHC Model for Dementia as coordinator, project manager, team leader, or other similar role.  Inclusion Criteria: Include statements related to engagement strategies and outcomes, e.g., how the formally appointed internal implementation leader became engaged with the Rural PHC Model for Dementia and what their role is in implementation. |
| 1. Champions | Definition: Individuals within Team 1 who dedicate themselves to supporting, marketing, and moving forward an implementation, overcoming indifference or resistance that the Rural PHC Model for Dementia may provoke in Team 1.  Inclusion Criteria: Include statements related to engagement strategies and outcomes, e.g., how the champion became engaged with the Rural PHC Model for Dementia and what their role is in implementation. |
| 1. External Change Agents | Definition: Individuals outside the health region who are affiliated with an outside entity who formally influence or facilitate Rural PHC Model for Dementia decisions in a desirable direction  Inclusion Criteria: Include statements related to engagement strategies and outcomes, e.g., how the external change agent (entities outside Team 1 that facilitate change) became engaged with the Rural PHC Model for Dementia and their role in implementation. |
| 1. Key Stakeholders | Definition: Individuals from within Team 1 that are directly impacted by the Rural PHC Model for Dementia, e.g., staff responsible for making referrals to a new program or using a new work process.  Inclusion Criteria: Include statements related to engagement strategies and outcomes, e.g., how key stakeholders became engaged with the Rural PHC Model for Dementia and what their role is in implementation. |
| 1. Innovation Participants | Definition: Individuals served by Team 1 that participate in the Rural PHC Model for Dementia, e.g., patients and families.  Inclusion Criteria: Include statements related to engagement strategies and outcomes, e.g., how Rural PHC Model for Dementia participants became engaged with the Model. |
| 1. Executing | Definition: Carrying out or accomplishing the implementation according to plan.  Inclusion Criteria: Include statements that demonstrate how implementation occurred with respect to the implementation plan. |
| 1. Reflecting & Evaluating | Definition: Quantitative and qualitative feedback about the progress and quality of implementation accompanied with regular debriefing about progress and experience.  Inclusion Criteria: Include statements that refer to the implementation team’s (lack of) assessment of the progress toward and impact of implementation, as well as the interpretation of outcomes related to implementation. |
| 1. **Additional Codes** |  |
| Innovation sustainability | Definition: Sustainability is concerned with the long-term term survival and effectiveness of the intervention [51].  Inclusion Criteria: Include statements that refer to continuation of the Rural PHC Model for Dementia by Team 1. |

*Note.* Construct definitions are adapted from the CFIR Codebook Template available on the CFIR website <https://cfirguide.org/tools/tools-and-templates/>
